# New Bills of Mortality for London

**Published:** 1840-07

**Authors:** 


					1840.] 289
PART FOURTH.
Jlftcirical SnteUtgetue*
TABLE OF MORTALITY FOR LONDON,
For the Fibst Quarter of 1840:
Showing the number of Deaths from all causes registered in twelve weeks, from the
5th January to the 28th March, 1840.
Causes of Death.
January.
Week ending
11th
18th 25th
February.
Week ending
1st 8th 15th 22d 29th
March.
Week ending
7th 14th 21st 28th
Total.
Class i.
Smallpox
Measles
Scarlatina
Hooping-cough
Croup
Thrush
Diarrhoea
Dysentery
Cholera
Influenza
Typhus
Erysipelas
Syphilis
Hydrophobia
Total Epidemic, &c.
Class it.
Cephalitis
Hydrocephalus
Apoplexy
Paralysis
Convulsions
Epilepsy
Insanity
Delirium tremens
Diseases of the brain, Sic..
Total Dis. of Brain, &c,
Class in.
Quinsey
Bronchitis
Pleurisy
Pneumonia
Hydro thorax
Asthma
Consumption
Diseases of the lungs, &c..
Total Dis. of Lungs, &c
Class iv.
Pericarditis
Aneurism
Diseases of the heart, &c...
Total Dis. of Heart, &c.
307
97
180
478
275
88
61
55
14
1
20
308
48
7
0
157
139
107
1632
133
415
231
187
680
58
20
16
121
156
178
170
1861
16
156
18
827
100
524
1740
201
373
302
276
263
276
270
3582
22
9
5
215
VOL. X. NO. XIX.
19
290 Medical Intelligence. [July.
Causes of Death.
January.
Week ending
11th 18th 25th
CLASS v.
Teething
Gastritis, enteritis ...
Peritonitis
Tabes mesenterica ...
Ascites
Ulceration
Hernia
Colic or ileus
Diseases of the stomach,&c.
Hepatitis
Jaundice
Disease of the Jiver, &c. ..
Total Dis.of Stomach, &c.
Class vi.
Nephritis
Diabetes
Stone
Stricture
Diseases of the kidneys, &c.
Total Dis. of Kidneys, &c.
Class vii.
Childbed
Ovarian dropsy
Diseases of uterus, &c..
Total Dis. of Uterus, &c
Class viii.
Rheumatism
Diseases of joints, &c. .
Total Dis. of Joints, &c
Classix.
Ulcer
Fistula
Diseases of skin, &c. .
Total Dis. of Skin, &c.
Class x.
Inflammation
Hemorrhage
Dropsy
Abscess
Mortification
Scrofula
Carcinoma
Tumour
Gout
Atrophy
Debility
Malformations
Sudden death
Total Dis. of Uncertain Seat.
Class xi.
Old age or natural decay ..
Class xu.
Intemperance
Privation
Violent deaths
Total by Violence, &c.
Class xui.
Causes not specified
34
as
76
21
21
February.
Week ending
1st 8th 15th 22d 29th
36
Total Deaths from all causes 967 997 916 835 818 813 855 916( 969 908 946 881 10821
99
78
19
19
58
5
0
31
4
4
2
9
1
0
4
17
o
13
119
65
16 24 36
128
March.
Week ending
7th 14th 21st 28th
84
48
47
115
75
34 25
67
27 24
Total.
1840.] Table of Mortality for London. 291
NUMBER OF DEATHS IN THE DIFFERENT DISTRICTS.
Districts.
Estimated Population
in 1840.
Deaths during
the Quarter.
West Districts
North Districts
Central Districts
East Districts
South Districts
308,921
414,458
369,722
411,634
450,265
1602
1990
2221
2345
2663
Total (Males 5510, Females 5311)
1,955,000
10,821
METEOROLOGICAL RESULTS FOR THE QUARTER.
Jan. Feb. March. Mean
Barometer   Highest
Lowest
Mean
Thermometer   Highest
Lowest
Mean
Dew point at 9 a. m  Highest
Lowest
Mean
Rain in Inches (Sum.)
30-56
29-31
29-75
54-2
22-3
40-2
47
17
37-0
30-59
28-64
29-87
51-0
27'8
39-4
42
23
34-9
30-65
29-74
30-19
53-7
30-9
38-8
40
25
33-5
29-81
39-7
35-6
Total.
2-63
1. Under the term London are comprised the thirty-two districts mentioned
in the next note, which include the City of London within and without the
walls, the City and Liberties of Westminster, the Out Parishes within the
bills of mortality; and the parishes of St. Mary-le-bone; St. Pancras; Ken-
sington; Fulham ; Hammersmith (Chapelry); St. Luke, Chelsea; Paddington ;
St. Mary, Stoke Newington; St. Leonard, Bromley; St. Mary-le-bow ; Cam-
berwell; Greenwich; St. Nicholas and St. Paul, Deptford; and Woolwich.
The population as enumerated in 1831 was 1,594,890.
2. West districts?Kensington; St. George, Hanover Square; Westminster;
St. Martin in the Fields; St. James. North districts?St. Mary-le-bone;
St. Pancras; Islington; Hackney. Central districts?St. Giles and St. George;
Strand; Holborn; Clerkenwell; St. Luke; East London; West London;
City of London. East districts?Shoreditch; Bethnal Green; Whitechapel;
St. George in the East; Stepney; Poplar. South districts?St. Saviour;
St. Olave; Bermondsey; St. George, Southvvark; Newington; Lambeth;
Camberwell; Rotherhithe ; Greenwich.
3. The diseases named in the subjoined list, when they occur, are included
in the tables under the titles which here follow them respectively :
Ague, Remittent Fever   Typhus.
Tetanus, Chorea, Myelitis, Lead Paralysis, Fright   Diseases of Brain.
Laryngitis   Diseases of Lungs.
Hypertrophy, Rupture of Aorta   Diseases of Heart.
Intussusception   Colic or Ileus.
Stricture of CEsophagus, Hematemesis, Diseases of Pan-
creas, Worms, Diseases of Rectum   Diseases of Stomach.
Diseases of Spleen   Diseases of Liver.
Ischuria, Cystitis   Diseases of Kidneys.
Paramenia, Abscess of Ovarium   Diseases of Utems.
Arthritis   Diseases of Joints.
292 Medical Intelligence. [July,
Carbuncle, Phlegmon, Scald Head    Diseases of Skin.
Purpura, Rickets  Scrofula.
Inflammation of the Absorbents, Dissection Wound .... Violent Deaths.
4. The Meteorological Results are taken from the journal kept at the apart-
ments of the Royal Society in London, and published monthly, in detail, in the
Athenaeum.

				

